# Predictors of endoscopic management failure in benign non-caustic esophageal strictures: a retrospective cohort study

**DOI:** 10.1016/j.clinsp.2026.101174

**Published:** 2026-10-15

**Authors:** Ilan David Friedmann, Sergio Szachnowicz, André Fonseca Duarte, Fauze Maluf-Filho, Rubens Sallum, Ulysses Ribeiro, Paulo Herman

**Affiliations:** Universidade de São Paulo, São Paulo, SP, Brazil

**Keywords:** Benign esophageal stricture, Endoscopic dilation, Esophagectomy, Predictors, Refractory stricture, Risk stratification

## Abstract

**Background:**

Benign Non-caustic Esophageal Strictures (BNES) often require repeated endoscopic dilations, yet a substantial proportion of patients remain refractory and ultimately require esophagectomy. Identifying predictors of endoscopic failure is essential for risk stratification and timely referral to alternative therapies. This study aimed to identify factors associated with endoscopic treatment failure leading to esophagectomy in patients with BNES.

**Methods:**

This retrospective cohort study included 51 patients with BNES treated at a single tertiary center from January 2004 to March 2026. Patients with caustic, malignant, or post-esophagectomy anastomotic strictures were excluded. Treatment consisted of balloon or bougie dilation. Endoscopic failure was defined as the need for esophagectomy due to persistent or recurrent dysphagia despite treatment. Multivariable logistic regression was performed to identify independent predictors of failure.

**Results:**

Of 51 patients, 18 (35.3%) required esophagectomy. Failure was associated with non-GERD etiology (p < 0.001), absence of hiatal hernia (p = 0.001), segmental strictures (p = 0.016), and a higher number of dilations (p = 0.026). In multivariable analysis, non-GERD etiology (OR = 7.45, p = 0.034) and absence of hiatal hernia (OR = 8.28, p = 0.016) were independent predictors. In a secondary model, absence of hiatal hernia (OR = 17.02, p = 0.011) and number of dilations (OR = 1.08 per session, p = 0.028) remained significant. Among endoscopically managed patients, 33.3% had persistent symptoms.

**Conclusions:**

One-third of BNES patients required esophagectomy. Non-GERD etiology, absence of hiatal hernia, and increasing dilation burden independently predict endoscopic failure and may support earlier consideration of surgical management.

## Highlights

• Endoscopic management fails in many patients with benign non-caustic strictures.
• Non-GERD etiology is a strong independent predictor of treatment failure.
• Absence of hiatal hernia is associated with a higher risk of esophagectomy.
• Increasing number of dilations reflects greater treatment refractoriness.
• Early risk stratification may support timely referral for surgical management.

## Introduction

Esophageal stricture is characterized by narrowing of the esophageal lumen, generally defined by a diameter <13–14 mm,^1^, ^2^, ^3^ a condition frequently associated with the development of dysphagia.^1^^,^^2^^,^^4^^,^^5^ In addition to this predominant symptom, patients with esophageal stricture may present chronic manifestations resulting from difficulty with oral intake, including malnutrition, weight loss, and impairment of quality of life.^4^^,^^5^ The causes of this condition are varied and include inflammatory, fibrotic, or neoplastic processes, which may directly affect the esophagus or result from alterations in adjacent structures.^1^^,^^4^

According to their etiology, esophageal strictures can be classified as benign or malignant.^6^ Among benign causes, the most notable are strictures secondary to ingestion of corrosive substances, peptic strictures associated with gastroesophageal reflux disease, radiation-induced strictures, eosinophilic esophagitis, drug-induced esophagitis, iatrogenic strictures following endoscopic therapies, thermal injuries, and anastomotic strictures, among others.^1^^,^^2^^,^^5^^,^^7^^,^^8^ Regardless of etiology, dysphagia is the main clinical manifestation and represents the primary therapeutic target.^1^^,^^2^

Benign Non-caustic Esophageal Strictures (BNES) represent a challenging clinical entity, often leading to significant dysphagia and impaired quality of life. These strictures can arise from various etiologies, including Gastroesophageal Reflux Disease (GERD), peptic injury, anastomotic complications (excluding post-esophagectomy), radiation therapy, eosinophilic esophagitis, and other inflammatory conditions.^1^^,^^2^ Endoscopic dilation remains the cornerstone of treatment, aiming to restore luminal patency and alleviate dysphagia. While generally effective, a substantial proportion of patients experience recurrence, requiring repeated dilations, or develop refractory strictures that do not respond to conventional endoscopic therapy.^3^^,^^4^

Currently, the treatment of benign esophageal strictures is initially based on endoscopic approaches. Endoscopic dilation of the stricture, performed with balloons or bougie dilators, remains the most widely used therapeutic method.^4^^,^^6^, ^7^, ^8^, ^9^ However, a proportion of patients show an unsatisfactory response to treatment. A stricture is defined as refractory when a luminal diameter ≥14 mm is not achieved or when dysphagia persists after three to five dilation sessions performed at two-week intervals.^3^^,^^4^^,^^9^ Recurrent strictures, in turn, are those in which there is a reduction of the lumen to values below 14 mm within a period of less than one month after a previously effective treatment.^3^^,^^9^

In cases of refractory or recurrent strictures, additional therapeutic strategies may be employed. These include dilation combined with intralesional corticosteroid injection, endoscopic incisional therapy for short strictures (generally <1 cm), and, in selected situations, the use of esophageal prostheses (stents) with the aim of maintaining luminal patency.^1^^,^^4^, ^5^, ^6^, ^7^, ^8^^,^^10^ These approaches are generally considered before the indication of surgical treatment^1^^,^^5^ (Fig. 1).

**Fig. 1:**
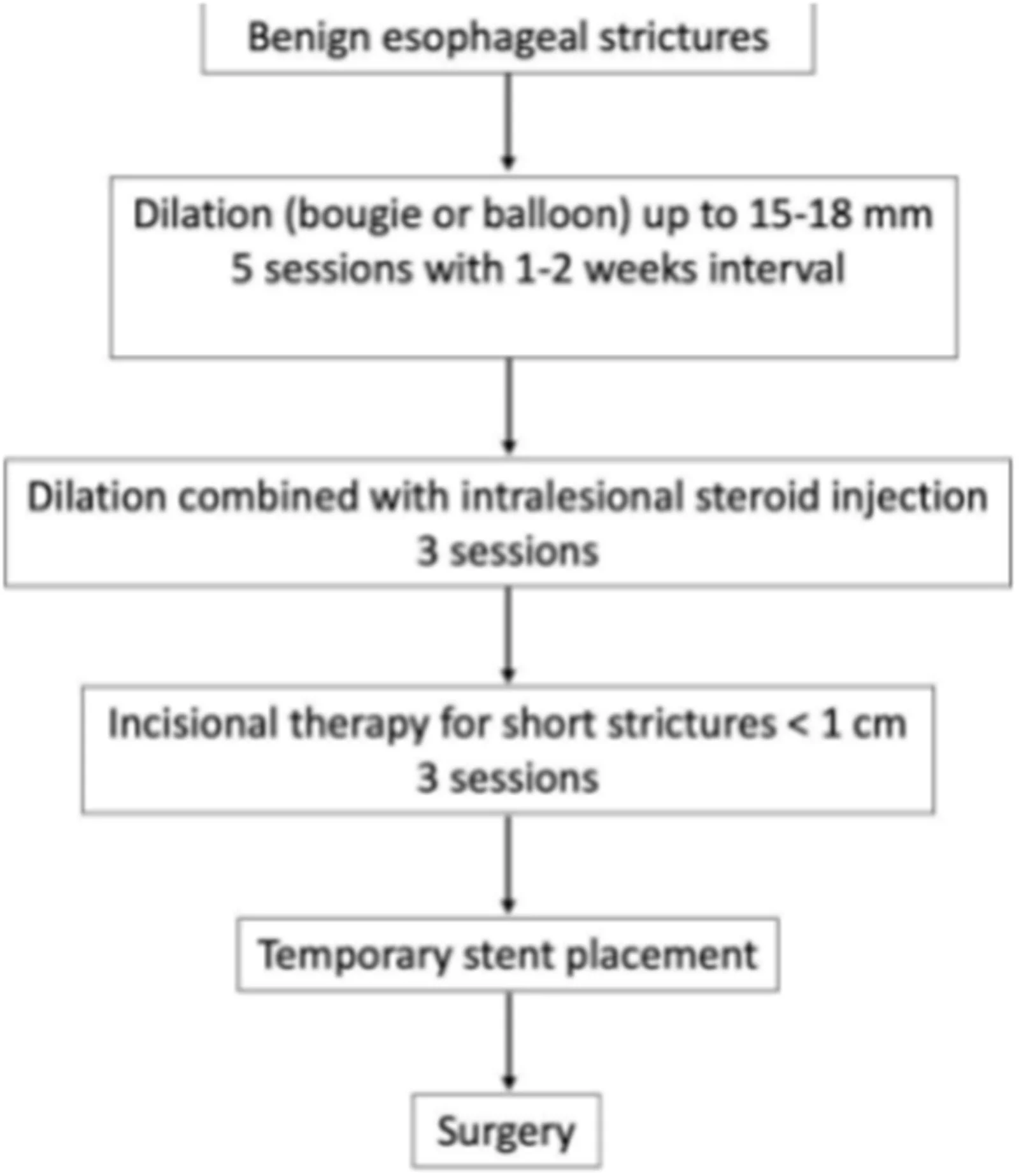
Stepwise management algorithm for benign esophageal strictures. Initial treatment consists of endoscopic dilation using bougie or balloon dilators up to 15‒18 mm, typically performed in up to five sessions at 1‒2 week intervals. In cases of persistent or recurrent strictures, dilation combined with intralesional steroid injection is recommended for three sessions. For short strictures (< 1 cm), incisional therapy may be performed in three sessions. If endoscopic approaches fail, temporary stent placement can be considered, with surgery reserved for refractory cases. Adapted from: Fugazza A, Repici A. Endoscopic management of refractory benign esophageal strictures. Dysphagia. 2021;36(3):504–516. doi:10.1007/s00455–021–10270-y.

Among benign strictures not related to caustic ingestion, the most frequent cause is peptic stricture secondary to gastroesophageal reflux disease.^1^^,^^2^ Several studies demonstrate that adequate control of esophageal acid exposure is directly associated with therapeutic success in these patients.^2^ This control can be achieved through pharmacological treatment with acid suppressants, mainly proton pump inhibitors or potassium-competitive acid blockers, or through antireflux surgery.^1^^,^^2^^,^^11^ Proper management of reflux may positively influence the outcomes of endoscopic treatment and reduce the need for more complex surgical interventions, such as esophagectomy.^1^^,^^2^^,^^11^

Despite the availability of different therapeutic strategies described in the literature, there is limited evidence to guide risk stratification and predict which patients will fail endoscopic therapy, especially in cases of inadequate response to endoscopic treatment.

This study aimed to identify independent predictors of endoscopic failure, defined as the need for esophagectomy, in patients with BNES managed at a single tertiary referral center. By analyzing a retrospective cohort, we sought to provide valuable insights into risk stratification that can inform clinical decision-making and improve patient outcomes and optimize the use of therapeutic resources, referring to surgery earlier the patients at higher risk of treatment failure.

## Methods

### Study design and population

This retrospective single-center cohort study was conducted at Hospital das Clínicas, Faculdade de Medicina da Universidade de São Paulo, a tertiary referral center in São Paulo, Brazil, through a systematic review of electronic and paper medical records, complemented by patient-reported information when available, from individuals treated.

All consecutive eligible patients followed in a specialized esophagus outpatient clinic with a diagnosis of benign esophageal stricture between January 2004 and March 2026 were included. Inclusion criteria were adult patients (≥ 18-years-old) with a diagnosis of benign non-caustic esophageal stricture who underwent at least one endoscopic dilation. Exclusion criteria included strictures of malignant etiology, caustic strictures, anastomotic strictures following esophagectomy, and patients with incomplete medical records precluding data extraction for key variables.

For primary analyses, patients were stratified into two groups: 1) Those who did not require esophagectomy during follow-up and 2) Those who underwent esophagectomy. Comparative analyses between groups were performed to identify clinical, epidemiological, and treatment-related factors associated with unfavorable outcomes.

Among patients managed without esophagectomy, a secondary analysis was performed to further classify individuals into two subgroups: 1) Those with documented clinical and/or endoscopic resolution of the stricture and 2) Those with persistent dysphagia or residual stricture at last follow-up. This subgroup analysis aimed to explore factors associated with incomplete endoscopic response.

Endoscopic treatment duration was defined as the interval, in months, between the first documented endoscopic dilation and the last dilation performed at our institution. For patients who underwent dilation at other institutions, treatment duration could not be reliably determined.

Follow-up time was defined as the interval between the first diagnostic evaluation performed at our center (upper gastrointestinal endoscopy or contrast esophagogram) and the last recorded assessment, esophagectomy, or the end of follow-up (March 2026), whichever occurred first.

This study was approved by the Institutional Review Board of the Hospital das Clínicas, Faculdade de Medicina da Universidade de São Paulo (approval n° 7.096.298; CAAE: 83,071,924.5.0000.0068) and was conducted in accordance with the Declaration of Helsinki. Given the retrospective design and use of medical records, patient consent was handled in accordance with institutional ethical guidelines.

This study was reported in accordance with the Strengthening the Reporting of Observational Studies in Epidemiology (STROBE) Statement.

### Data collection

Demographic data (age, sex, Body Mass Index [BMI] and smoking history), clinical characteristics (stricture etiology, presence of hiatal hernia, stricture length, stricture type ‒ punctiform vs. segmental), and treatment details (number of dilation sessions, type of dilator used, use of intralesional steroids) were extracted from electronic medical records. Stricture etiology was categorized as GERD-related (peptic) or non-GERD (e.g., radiation-induced, post-surgical, inflammatory, idiopathic). Stricture length was determined from endoscopic reports when measurements were documented by the endoscopist. When endoscopic measurements were unavailable, length was extracted from contrast esophagography (barium swallow) reports. Segmental strictures were defined as those longer than 1 cm, whereas punctiform strictures were defined as those measuring ≤ 1 cm, according to previously published criteria.^12^ Only one patient had multiple stricture sites, both measuring ≤ 1 cm, therefore, the patient was classified as having punctiform strictures. Variables related to gastroesophageal reflux management, including prior use of proton pump inhibitors and history of fundoplication, were collected for descriptive purposes.

### Endoscopic management protocol

Endoscopic dilations were performed using either Savary-Gilliard bougies or Through-The-Scope (TTS) balloon dilators. The choice of dilator was at the discretion of the endoscopist. Dilation was typically initiated with a small-diameter dilator and progressively increased in size, usually by 1‒2 mm increments per session, aiming for a target diameter of 15‒18 mm. Dilation sessions were typically spaced 2‒4 weeks apart. Intralesional triamcinolone injections (e.g., 40 mg in 1 mL, divided into 4 quadrants) were optionally used in cases of refractory strictures (defined as recurrence within 4-weeks after 3‒5 dilations to 14 mm) at the discretion of the treating physician.

### Definition of endoscopic failure and outcomes

The primary outcome was endoscopic failure, defined as the eventual need for esophagectomy due to persistent dysphagia despite repeated endoscopic dilations. Criteria for esophagectomy were based on individualized assessments during multidisciplinary meetings involving digestive surgeons, endoscopists, gastroenterologists, and nutritionists. Patients with refractory strictures, particularly those experiencing persistent difficulty in maintaining nutritional status, significant impairment in quality of life, or inability of endoscopic treatment to sustain a pasty diet for more than two weeks, were considered for surgical intervention.

Secondary outcomes included the proportion of patients achieving stricture resolution in the endoscopically managed group, defined as sustained dysphagia relief (grade 0‒1 on a 0‒4 scale) with preserved body weight and nutritional status, the ability to maintain a pasty diet for > 2-weeks without additional dilations, and absence of significant luminal narrowing on follow-up endoscopy or esophagogram, as well as overall follow-up duration.

### Statistical analysis

Statistical analyses were performed using JASP software (version 0.96.0; JASP Team, University of Amsterdam, The Netherlands).

Descriptive statistics were used to summarize patient characteristics. Continuous variables were assessed for normality using the Shapiro-Wilk test and are presented as mean ± Standard Deviation (SD) or median (Interquartile Range [IQR]), as appropriate. Group comparisons were performed using the Student’s *t*-test or Mann-Whitney *U test*, accordingly.

Categorical variables, including sex, smoking status, etiology (peptic vs. non-peptic), presence of hiatal hernia, Barrett’s esophagus, stricture length (punctiform vs. segmental), and type of reflux management (medical therapy alone vs. combined medical and surgical treatment), are presented as absolute and relative frequencies. For primary analyses, comparisons were performed using the Chi-Square test or Fisher’s exact test, as appropriate. In subgroup analyses involving small sample sizes, categorical variables were compared using Fisher’s exact test.

Multivariable logistic regression analysis was performed to identify factors independently associated with failure of endoscopic treatment (esophagectomy). Variable selection was based on clinical relevance and univariable analysis (p < 0.10).

A primary model included baseline variables (etiology, presence of hiatal hernia, and stenosis extension). A secondary model replaced stenosis extension with the number of dilations to account for treatment burden.

Results are presented as Odds Ratios (ORs) with 95% Confidence Intervals (95% CIs). Model discrimination was assessed using the Area Under the receiver operating Characteristic Curve (AUC), and calibration using the Hosmer-Lemeshow goodness-of-fit test.

Confidence intervals for proportions were calculated using the Wilson method. All tests were two-sided, and a p-value < 0.05 was considered statistically significant.

## Results

A total of 51 patients with benign non-caustic esophageal strictures were included in the study. The mean age was 44.9 ± 20.3 years, and 58.8% were male. During the follow-up period, 18 patients (35.3%) ultimately required esophagectomy due to endoscopic failure, while 33 patients (64.7%) were successfully managed endoscopically.

### Baseline characteristics

Table 1 summarizes the baseline characteristics of patients stratified by treatment outcome. There were no significant differences in age, sex, BMI, or smoking status between the two groups. Patients who underwent esophagectomy had a significantly higher prevalence of non-GERD etiology (50%vs. 6.3%, p < 0.001) and absence of hiatal hernia (82.4%vs. 33.3%, p = 0.001). Segmental strictures were also more common in the esophagectomy group (58.8%vs. 24.2%, p = 0.016). The median number of dilation sessions was significantly higher in the esophagectomy group (15 sessions, IQR 4‒39) compared to the endoscopic success group (5 sessions, IQR 2‒8.5) (p = 0.026). Information regarding intralesional triamcinolone use was available for 48 patients. Among these, steroid injection was documented in two of 30 patients in the endoscopic success group and four of 17 patients in the esophagectomy group. No patients underwent endoscopic incisional therapy or esophageal stent placement. The median follow-up duration for the endoscopic success group was 70-months (IQR 27‒137), significantly longer than the 15-months (IQR 6‒36.3) for the esophagectomy group (p = 0.005) (Table 1).

**Table 1:** Baseline characteristics of all patients by treatment outcome (endoscopic success vs. esophagectomy).

Characteristic	Endoscopic Success (n = 33)	Esophagectomy (n = 18)	p-value
Age, mean ± SD	46.8 ± 20.7	41.4 ± 19.5	0.369
Sex, n (%) female	13/33 (39.4%)	7/18 (38.9%)	0.920
Smoking	13/33 (39.4%)	4/16 (25%)	0.526
Hiatal hernia	22/33 (66.7%); 95% CI: (49.6‒80.2)	3/17 (17.6%); 95% CI (6.2‒41.0)	0.001
GERD etiology	30/32 (93.7%); 95% CI: (79.9‒98.3)	9/18 (50%); 95% CI: (29.0‒71.0)	<0.001
Segmental strictures	8/33 (24.2%); 95% CI: (13.6‒39.4)	10/17 (58.8%); 95% CI: (36.0‒78.4)	0.016
Median number of dilations (IQR)	5.0 (2.0‒8.5), n = 32	15.0 (4.0‒39.0), n = 17	0.026
intralesional triamcinolone use	2/30 (6.7%); 95% CI: (1.8%‒21.3%)	4/17 (23.5%); 95% CI: (9.6‒47.3)	0.170
Median follow-up (months, IQR)	70.0 (27.0‒137.0)	15 (6.0‒36.2)	0.005

Note: Data are presented as mean ± Standard Deviation (SD), median (interquartile range [IQR]), or n/N (%). Denominators vary due to missing data. Continuous variables were compared using the Mann-Whitney *U test*, and categorical variables using the chi-square or Fisher’s exact test, as appropriate. A two-sided p-value < 0.05 was considered statistically significant.

Missing values: smoking (n = 2), hiatal hernia (n = 1), GERD etiology (n = 1), stricture type (n = 1), number of dilations (n = 2) and intralesional triamcinolone use (n = 1) in the esophagectomy group; GERD etiology (n = 1), number of dilations (n = 1) and intralesional triamcinolone use (n = 3) in the endoscopic success group.

### Predictors of endoscopic failure

Two multivariate logistic regression models were developed to identify independent predictors of endoscopic failure (Table 2). The primary model included stricture etiology, presence of hiatal hernia, and stricture type. In this model, non-GERD etiology (OR = 7.45, 95% CI 1.16‒47.94, p = 0.034) and absence of hiatal hernia (OR = 8.28, 95% CI 1.49‒46.03, p = 0.016) were significant independent predictors of requiring esophagectomy. Segmental stricture showed a trend towards significance (OR = 4.02, 95% CI 0.78‒20.72, p = 0.096). The AUC for this model was 0.867, indicating good discriminative ability.

**Table 2:** Multivariable logistic regression analysis for predictors of esophagectomy.

Predictor	Odds Ratio (OR)	95% Confidence Interval	p-value
Model 1: Etiology, Hiatal Hernia, Stricture Type (AUC = 0.867)			
Non-GERD Etiology	7.45	1.16–47.94	0.034
Absence of Hiatal Hernia	8.28	1.49–46.03	0.016
Segmental Stricture	4.02	0.78–20.72	0.096
Model 2: Hiatal Hernia, Number of Dilations (AUC = 0.924)			
Absence of Hiatal Hernia	17.02	1.90–152.30	0.011
Number of Dilations (per dilation)	1.08	1.01–1.16	0.028

Note: Model 1 includes baseline variables (etiology, presence of hiatal hernia, and stricture length). Model 2 replaces stricture length with the number of endoscopic dilations to reflect treatment burden. Odds Ratios (ORs) are presented with 95% Confidence Intervals (95% CIs).

A secondary model was constructed to assess the impact of the number of dilations, given its strong association in univariate analysis. This model included absence of hiatal hernia and number of dilations. In this model, absence of hiatal hernia remained a strong predictor (OR = 17.02, 95% CI 1.90‒152.30, p = 0.011), and a higher number of dilations was also independently associated with endoscopic failure (OR = 1.08 per dilation, 95% CI 1.01‒1.16, p = 0.028). Non-GERD etiology was not statistically significant in this model. The AUC for this secondary model was 0.924, suggesting excellent predictive performance (Table 2).

Within the endoscopic group, 11 patients (33.3%) had persistent stricture or dysphagia at last follow-up, while 22 (66.7%) achieved complete clinical resolution. No significant differences were observed between these groups regarding demographic characteristics, clinical variables, or treatment parameters (Table 3). However, patients with persistent symptoms showed a non-significant trend toward a higher prevalence of segmental strictures and a lower number of dilation sessions, suggesting a potential association between stricture complexity and incomplete endoscopic response (Table 3).

**Table 3:** Secondary analysis: persistent vs. resolved endoscopic group.

Characteristic	Persistent (n = 11)	Resolved (n = 22)	p-value
Age, mean ± SD	45.0 ± 20.57	47.68 ± 21.23	0.732
Sex, n (%) female	6 (54.6%)	7 (31.8%)	0.270
Smoking, n (%)	3 (27.3%)	9 (40.9%)	0.703
Hiatal hernia, n (%)	5 (45.6%)	17 (77.3%)	0.117
GERD etiology, n (%)	10/10 (100%)	20 (90.9%)	1.000
Segmental strictures, n (%)	4 (36.4%)	4 (18.2%)	0.391
Median number of dilations (IQR)	3 (2.0‒6.5)	6 (2.0‒10.0)	0.689
Median follow-up (months, IQR)	70.0 (10.0‒146.0)	62.0 (33.0‒130.3)	0.804

Note: Data are presented as mean ± Standard Deviation (SD), median (interquartile range [IQR]), or n/N (%), as appropriate. Percentages were calculated based on available data for each variable. Continuous variables were compared using the Mann-Whitney *U test*. All categorical variables were analyzed using Fisher’s exact test due to the small sample size, which may lead to low expected cell counts and violate chi-square assumptions. All tests were two-sided, and a p-value < 0.05 was considered statistically significant. Given the small sample size, these subgroup comparisons should be considered exploratory and interpreted with caution.

## Discussion

In this retrospective cohort of patients with non-caustic benign esophageal strictures, approximately one-third of patients (35.3%) ultimately required esophagectomy despite endoscopic management.

We identified key independent predictors of endoscopic failure leading to esophagectomy, including non-GERD etiology, absence of hiatal hernia, and a higher number of endoscopic dilations. Segmental strictures also showed a consistent, although not statistically significant, association with worse outcomes.

To our knowledge, few studies have specifically evaluated predictors of esophagectomy in this population, and none have clearly integrated clinical and treatment-related factors into a risk stratification framework as presented here.

Our results reinforce the concept that etiology plays a central role in the natural history and treatment responsiveness of benign esophageal strictures. Peptic strictures, typically associated with chronic gastroesophageal reflux, tend to respond more favorably to dilation combined with acid suppression, whereas non-GERD etiologies, such as post-radiation, post-surgical, or inflammatory causes, often involve deeper fibrosis and impaired tissue remodeling, which may explain their higher likelihood of progression to esophagectomy.^13^, ^14^, ^15^, ^16^ These results are consistent with prior studies suggesting that non-peptic strictures represent a more refractory phenotype, and this difference in underlying pathology likely explains the higher rate of endoscopic failure in this group.

The absence of a hiatal hernia emerged as another significant predictor of endoscopic failure. While seemingly counterintuitive, as hiatal hernia is a common predisposing factor for GERD and peptic strictures, its absence may indicate a different, more complex underlying mechanism for stricture formation. In patients with GERD-related strictures, the presence of a hiatal hernia often serves as a marker for chronic reflux, which, when adequately managed, can lead to better outcomes from dilation.^15^, ^16^, ^17^, ^18^, ^19^ Conversely, in the absence of a hiatal hernia, a stricture might be indicative of a more severe or atypical form of reflux, or a non-reflux underlying etiology that is inherently more difficult to treat endoscopically. This suggests that hiatal hernia may act as a surrogate marker for a more “favorable” peptic phenotype that responds better to standard endoscopic and medical or surgical therapies for GERD, rather than an independent protective factor.

The role of treatment burden is also clinically relevant. In our secondary model, the number of dilations was independently associated with esophagectomy, with each additional session increasing the odds of surgical intervention. The number of dilations required is a direct reflection of the stricture's refractoriness. Our finding that a higher number of dilations independently predicts the need for esophagectomy underscores the importance of recognizing when endoscopic therapy is reaching its limits.

While there is no universally agreed-upon threshold, persistent dysphagia after ≥ 5 dilations and recurrence within less than one month are consistent with established definitions of refractory and recurrent benign esophageal strictures.^5^ Nonetheless, from a pragmatic perspective, these findings suggest that repeated need for dilation may serve as an early warning signal of treatment failure,^20^ potentially supporting earlier referral to surgical evaluation in selected patients. Consistent with Chaibi et al. (2025), who in a series of 46 patients identified peptic origin (OR = 6) and higher dilations (OR = 2.2) as recurrence predictors, our study further supports the cumulative burden of dilations as a marker for eventual surgical intervention.^21^

Although segmental strictures did not reach statistical significance in multivariable analysis, it consistently showed a substantial effect size, suggesting a clinically meaningful association. The lack of statistical significance is likely related to the limited sample size and wide confidence intervals. From a clinical standpoint, longer strictures are generally more challenging to manage endoscopically, often requiring repeated interventions and, in selected cases, advanced endoscopic techniques such as incisional therapy.^12^ This is consistent with the observed trend toward worse outcomes in patients with segmental disease.

For patients identified as high-risk based on these predictors (e.g., non-GERD etiology, absence of hiatal hernia, and/or requiring a high number of dilations), a more aggressive or alternative approach may be warranted. This could include earlier consideration of advanced endoscopic techniques such as endoscopic incisional therapy for short, web-like strictures (< 1 cm), temporary placement of fully covered Self-Expandable Metal Stents (SEMS) or biodegradable stents, or intralesional steroid injections.^6^^,^^7^^,^^16^ For those with multiple high-risk factors, early referral for surgical evaluation, including the potential for esophagectomy, might be a more appropriate and patient-centered approach, avoiding prolonged and ultimately futile endoscopic interventions.

Among patients managed without esophagectomy, no clear predictors of incomplete clinical resolution were identified. However, there was a trend toward more complex (segmental) strictures and fewer dilation sessions among those with persistent symptoms. This observation raises the possibility that, in some cases, insufficient treatment intensity or early discontinuation of dilation may contribute to suboptimal outcomes, although this hypothesis requires further investigation.

Clinically, patients with non-GERD etiology, absence of hiatal hernia, and increasing dilation requirements represent a high-risk subgroup for endoscopic failure. Early recognition of these features should prompt closer follow-up and consideration of earlier surgical evaluation. The decision-making process can be guided by a risk stratification algorithm as depicted in Fig. 2.

**Fig. 2:**
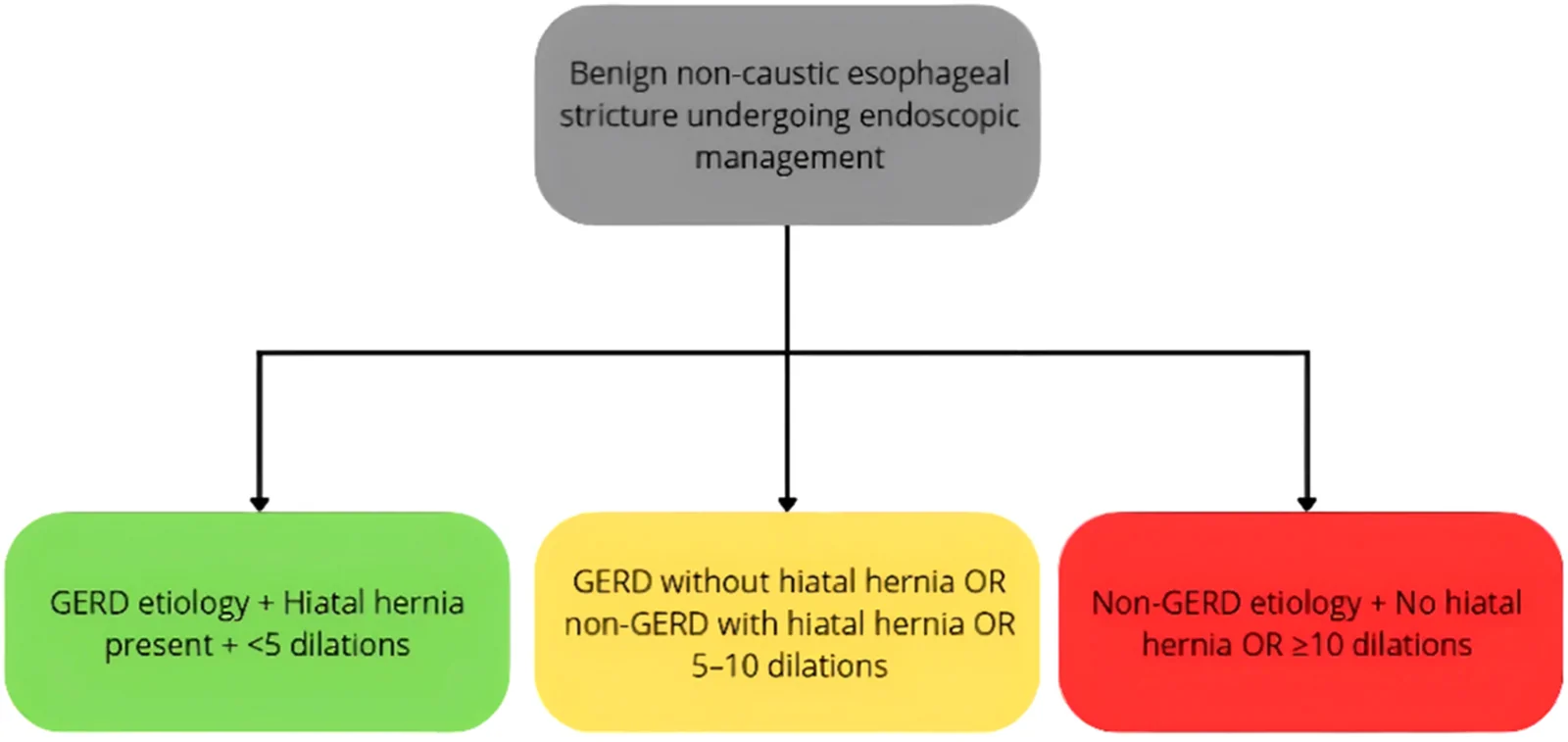
Proposed risk stratification and management algorithm for benign non-caustic esophageal strictures.

### Limitations

This study has several limitations. First, its retrospective, single-center design introduces the possibility of selection bias and may limit the generalizability of the findings. Second, the relatively small sample size reduced statistical power, particularly in subgroup analyses, and resulted in wide confidence intervals for some odds ratios; therefore, these estimates should be interpreted cautiously and validated in larger cohorts.

The retrospective nature of the study introduces the potential for missing data, and exclusion of patients with incomplete records may have introduced selection bias. Intralesional steroid injection was infrequently documented (n = 6), precluding meaningful assessment of its independent effect on treatment outcomes. In addition, the decision to proceed with esophagectomy was based on multidisciplinary clinical assessment rather than a standardized protocol, which may have introduced variability in treatment selection. Follow-up duration was longer in the endoscopic success group, potentially increasing the opportunity to detect late recurrences; however, the primary outcome was esophagectomy, which reduces the impact of this difference on the main findings. Furthermore, detailed information on specific non-GERD etiologies was insufficient to permit meaningful subgroup analyses. The secondary analysis of persistent symptoms among endoscopically managed patients was limited by the small sample size and was likely underpowered to detect clinically relevant predictors; therefore, these findings should be interpreted cautiously and require validation in larger studies. Future prospective multicenter studies with larger cohorts are needed to validate these findings and further refine risk stratification models.

## Conclusion

Non-GERD etiology, absence of hiatal hernia, and a higher number of endoscopic dilations are significant independent predictors of endoscopic failure requiring esophagectomy in patients with benign non-caustic esophageal strictures. These findings highlight the importance of careful patient selection and early risk stratification. Identifying high-risk patients based on these factors can facilitate timely consideration of alternative endoscopic therapies or early surgical referral, potentially improving outcomes and reducing the burden of prolonged, ineffective endoscopic management.

These findings suggest that clinical and anatomical characteristics can help identify patients at higher risk of failure of conservative therapy. In particular, repeated need for dilation may serve as a practical indicator of refractory disease, supporting closer monitoring and earlier consideration of surgical evaluation.

Overall, this study provides real-world evidence to support risk stratification in benign esophageal strictures and may contribute to more individualized and timely therapeutic decision-making. Future prospective studies are warranted to validate these findings and refine predictive models for clinical use.

## Funding

FAPESP ‒ Fundação de Amparo à Pesquisa do Estado de São Paulo.

## Conflicts of interest

The authors declare no personal, financial, or institutional interest in any of the drugs, materials, or devices described in this article.

## Data Availability

The data are available from the corresponding author upon reasonable request.
